# Readiness to Change among Adolescents with Chronic Pain and Their Parents: Is the German Version of the Pain Stages of Change Questionnaire a Useful Tool?

**DOI:** 10.3390/children7050042

**Published:** 2020-05-02

**Authors:** Lorin Stahlschmidt, Susanne Grothus, Donnamay Brown, Boris Zernikow, Julia Wager

**Affiliations:** 1 German Paediatric Pain Centre, Children’s and Adolescents’ Hospital, 45711 Datteln, Germany; L.Stahlschmidt@deutsches-kinderschmerzzentrum.de (L.S.); S.Grothus@deutsches-kinderschmerzzentrum.de (S.G.); D.Brown@deutsches-kinderschmerzzentrum.de (D.B.); B.Zernikow@kinderklinik-datteln.de (B.Z.); 2 Department of Children’s Pain Therapy and Paediatric Palliative Care, Faculty of Health, School of Medicine, Witten/Herdecke University, 58448 Witten, Germany

**Keywords:** pain management, chronic pain, adolescents, stages of change, PSOCQ, intensive interdisciplinary pain treatment

## Abstract

The Pain Stages of Change Questionnaire (PSOCQ) measures patients’ willingness to engage in active self-management of their pain. The present study aimed to create validated German short versions of the PSOCQ for adolescents (PSOCQ-A) and their parents (PSOCQ-P). Additionally, an investigation of stages of change regarding pain characteristics and treatment outcomes was undertaken. In Study 1, the data of adolescents aged 11 to 18 years and their parents were collected prior to intake (*N* = 501) and at admission (*N* = 240) to specialist inpatient pain treatment. Confirmatory factor analyses indicated a poor fit of the full PSOCQ measures prior to intake, but an acceptable fit at admission. Short PSOCQ-A and PSOCQ-P versions were identified. In Study 2, these results were cross-validated with data from an additional *N* = 150 patients and their parents, collected during and 3 months after interdisciplinary inpatient pain treatment. Model fits for both short versions were acceptable, although low internal consistency for the PSOCQ-A Precontemplation and Contemplation subscales was identified. During treatment, both patients’ and their parents’ readiness to change increased. Stage of change at discharge did not predict treatment non-response 3 months later. This study indicates that the PSOCQ is neither meaningful prior to admission nor predictive of non-response to treatment. While some value may exist in monitoring treatment progress, based on the results of this study, it is not recommended that the PSOCQ-A and PSOCQ-P be used as a measure of stage of change in German pediatric pain populations.

## 1. Introduction

Interdisciplinary pain treatment programs for highly impaired adolescents with severe chronic pain usually take an active self-management approach [1]. This means that patients are actively involved in, and personally responsible for, managing their pain by applying active pain coping strategies. Prior to treatment, most patients believe medical interventions will manage their condition [2], however, during interdisciplinary pain treatment, they learn the skills required to manage their own pain [1]. Insufficient motivation to self-manage pain is thought to be an important factor contributing to unsatisfactory treatment outcomes in both pediatric and adult pain patients [3,4,5,6].

The Pain Stages of Change Questionnaire (PSOCQ), originally developed for adults [7], is derived from the transtheoretical model [8], which postulates that individuals experience differing levels of willingness to employ pain self-management strategies in identifiable stages. Four stages of change are assessed by the PSOCQ; Precontemplation, Contemplation, Action and Maintenance [7]. Briefly, in Precontemplation, patients typically lack personal responsibility for their pain management, looking towards medical interventions for solutions to their pain. Contemplation is characterized by awareness of the need to change pain coping [2]. The Action stage is identifiable by the recent commencement of self-management strategies, and Maintenance occurs when the patient routinely uses pain self-management strategies in everyday life [2]. 

Guite et al. [2] adapted the adult version of the PSOCQ for use in adolescents with chronic pain (PSOCQ-A). As parents are usually involved in their child’s pain management, a parent version was also created (PSOCQ-P). Factor analyses combined the Action and Maintenance stages within the PSOCQ-A [2]. Thus, for adolescents, the questionnaire consists of three, instead of four, stages of change. For parents, four stages of change were confirmed [2]. Although a German version of the PSOCQ for adults exists [9], to date, no German versions of the PSOCQ-A and PSOCQ-P are available. Therefore, the overarching aim of the present study was to create validated German versions of the PSOCQ-A and the PSOCQ-P. A previously conducted pilot study revealed low acceptance of both German questionnaires [10] because participants had difficulty interpreting items, particularly prior to specialized pain treatment in which patients often learn about the concept of active pain self-management for the first time. Therefore, determining the temporal utility of the questionnaire during different stages of treatment was identified as important. To address these goals, two studies were conducted. The aims of Study 1 were to investigate the factor structure of the PSOCQ-A and PSOCQ-P, to create short versions of the questionnaires to increase usability in clinical practice, and to investigate the suitability of the PSOCQ at different stages prior to treatment. The aims of Study 2 were to cross-validate the short versions developed in Study 1 in an independent sample. Sensitivity to change at different time points during and after treatment was investigated, as was criterion validity, by means of associations between readiness to change, pain characteristics and treatment outcomes.

## 2. Materials and Methods

### 2.1. Study 1

#### 2.1.1. Participants, Setting and Procedure

Adolescents and their parents attending the German Paediatric Pain Centre between September 2015 and February 2017 were eligible to participate. Data were collected from *N* = 501 adolescents and their parents (*N* = 407) during their initial intake assessment (Sample A). Surveys were completed by participants as part of the standard diagnostic pack sent out to families prior to this first consultation. 

Additionally, during the same data collection period, data from *N* = 240 adolescent patients, and their parents (*N* = 224), who were attending an admission appointment for the three-week intensive interdisciplinary pain treatment (IIPT) were collected (Sample B). This pain treatment program is an in-patient program designed to treat children and adolescents with functionally restricting pain, if indicated. All patients aged 11 to 18 years able to complete the questionnaires themselves were eligible to participate. Adolescents and parents who had missing PSOCQ data were excluded from analyses. Table 1 displays the participant characteristics of the study samples.

#### 2.1.2. Measures

*Readiness to change*: Motivation to self-manage pain was self-reported using the German versions of the PSOCQ-A and the PSOCQ-P. Both versions were translated into German in a forward-backward process [11]. Both PSOCQ versions consist of 30 items that are assigned to one of the four stages of change: Precontemplation (seven items), Contemplation (10 items), Action (six items) and Maintenance (seven items). The parent version mirrors the adolescent version. Answers are given on a five-point Likert scale ranging from 1 (“I strongly disagree”) to 5 (“I strongly agree”). For the English version of the PSOCQ-A, a three-factor structure was found with a combined Action/Maintenance scale [2,9]. The PSOCQ-P had a four-factor structure [2,10]. Internal consistencies for the subscales ranged from 0.67 to 0.93, with the lowest values found for the Precontemplation subscale [2,11].

*Pain intensity*: Average pain intensity in the past four weeks was assessed with a numerical rating scale (NRS) from 0 = no pain to 10 = strongest pain. The NRS enables a reliable and valid measure of pain intensity in adolescents [12,13,14].

*Functional impairment*: Functional impairment was assessed with measures of pain-related school absence, i.e., the number of missed school days due to pain, and pain-related disability in daily activities, assessed with the Pediatric Pain Disability Index (PPDI; [15]). Sample A reported numbers of missed school days due to pain in the previous three months; Sample B in the previous four weeks. For comparability, school absence reported by Sample A was divided by three, resulting in an approximate value for the last four weeks. The PPDI enables a valid assessment of interference due to pain with 12 daily activities, such as partaking in school, meeting friends or participating in sports on a five-point Likert scale (1 = never, 5 = always) [15,16]. The total score ranges from 12 to 60, with higher values indicating more severe disability. The PPDI demonstrated the internal consistency of Cronbach’s α = 0.86 to 0.87 [15,16]. For each patient, up to two missing values were imputed with the patient’s mean.

#### 2.1.3. Ethics

Ethical approval for the two studies reported in this paper was obtained from the ethics committee of the children’s hospital. A positive vote was provided on 2 August 2017 (reference number: 2017/08/02 BZ2). Because data were collected as a component of the standard clinical diagnostic procedure, a waiver for need of informed consent was granted. 

#### 2.1.4. Statistical Analyses

All data analyses were performed in AMOS and SPSS (release 25.0 for Windows). To test the factor structure and the suitability of the PSOCQ-A and PSOCQ-P for a more general pain sample prior to any treatment (Sample A) and a more clinically homogenous pain sample who had been informed of pain psychoeducation (Sample B), confirmatory factor analyses for both samples were conducted in AMOS using maximum likelihood estimation. The following conventions for model fit indices were applied: χ^2^/df (≤3 = acceptable, ≤2 = good), Comparative Fit Index (CFI; ≥0.95 = acceptable, ≥0.97 = good), Root Mean Square Error of Approximation (RMSEA; ≤0.08 = acceptable, ≤0.05 = good), and Standardized Root Mean Square Residual (SRMR; ≤0.10 = acceptable, ≤0.05 = good) [17]. We tested the original four-factor (Precontemplation, Contemplation, Action, Maintenance) and a three-factor model (Precontemplation, Contemplation, combined Action/Maintenance) for both PSOCQ-A and PSOCQ-P [2,18]. Cronbach’s α was used as a measure of the internal consistencies of the subscales. To shorten both PSOCQ versions, items with factor loadings of < 0.5 were deleted from the scales; model fit and internal consistency were reevaluated for the shortened versions.

### 2.2. Study 2

#### 2.2.1. Participants and Procedure

The Study 2 sample consisted of *N* = 150 adolescents who received IIPT at the pain center between July 2017 and May 2018. All patients aged 11 to 18 years who were able to self-report in questionnaires were eligible for the study. As Study 1 demonstrated that readiness to change was not meaningfully measured prior to admission, in Study 2, we only collected data during IIPT, when patients and their parents were familiar with the pain self-management concept. Data were collected at five timepoints: T1 = admission; T2 = one week after admission; T3 = two weeks after admission; T4 = discharge (approximately three weeks after admission); T5 = three months after discharge. Patients with missing PSOCQ-A data collected during admission were excluded from analyses. 

Additionally, data from parents were collected at T1 (*N* = 150), T4 and T5. For T1 to T4, data were collected as part of the standard diagnostic procedure during the inpatient stay at the pain center. For the follow-up assessment (T5), patients and parents were sent the questionnaires along with the study information and an informed consent form by mail. They were asked to return the informed consent form and the questionnaires in a prepaid return envelope. If they did not return the questionnaires within two weeks, they were contacted via telephone and asked whether they would be willing to participate. Overall, *N* = 127 patients (84.7%) participated at T5. Table 1 displays the participant characteristics of Study 2.

#### 2.2.2. Measures

*Readiness to change*: The short form of the PSOCQ-A was used for all data collection timepoints. Parents completed the short form of the PSOCQ-P at T1, T4 and T5. 

*Pain intensity*: At T1 and T5, numerical rating scales (NRS) from 0 = no pain to 10 = strongest pain were used to assess maximum and average pain intensity in the last four weeks. 

*Functional impairment*: Patients reported the number of school days absent due to pain in the past four weeks and pain-related disability in daily activities with the PPDI [15] at T1 and T5. 

*Pain coping:* The Pediatric Pain Coping Inventory revised (PPCI-r; [19]) was used to assess pain coping at T1. The measure consists of 25 items which assess three different coping styles: passive pain coping (10 items), seeking social support (eight items) and positive self-instruction (seven items). Patients indicate how often they use different coping strategies on a three-point Likert scale from 0 = almost never to 2 = frequently. A validation study has noted acceptable internal consistency, with Cronbach’s α of the scales ranging from 0.71 to 0.80 [19].

#### 2.2.3. Ethics

The ethics committee of the children’s hospital approved the study. Data collected during the inpatient stay were part of the standard diagnostic procedure, and the study was granted a waiver for the need of informed consent for these data. For the follow-up assessment at T5, patients and their parents provided informed consent.

#### 2.2.4. Statistical Analyses

All data analyses were performed in AMOS and SPSS (release 25.0 for Windows). For cross-validation of the factor structure of the PSOCQ short-versions developed in Study 1 (see Appendix A for allocation of items and factors), CFAs were conducted with T1 (admission) data. Only adolescent (*N* = 150) and parent (*N* = 142) data without missing PSOCQ values were included in these analyses. Cronbach’s alphas (α) were calculated to measure internal consistency for each subscale.

Consistent with Guite et al. [2], patients were assigned to one stage of change based on their highest subscale mean score. In the case where two mean scores were equal, the patient was assigned to the more advanced stage. Sensitivity to change over subsequent timepoints was assessed with Friedman and Sign tests for ordinal data.

In lieu of a comparable measure or ‘gold standard’ measure of stages of change, criterion validity was measured by assessing associations with pain-related outcomes to determine whether the measure responded to treatment outcome scores in a manner consistent with expected outcomes. Concurrent associations with pain characteristics were analyzed with Kruskal–Wallis tests with the stage of change assigned at T1 as the independent variable and the T1 pain characteristics (pain intensity, pain-related disability in daily activities, pain-related school absence, pain coping) as the dependent variables. Mann–Whitney-U-tests were conducted as post-hoc analyses for significant overall tests. Worse pain-related outcomes were expected in earlier stages of change [18]. Predictive criterion validity was assessed by assessing associations between stages of change during treatment with pain-related outcomes at discharge and three-months post-treatment (T5). Our primary outcome was treatment response measured by a compound measure of pain severity. The Chronic Pain Grading (CPG) integrates pain intensity and functional impairment into one measure of overall pain severity. Patients are assigned to one of five pain severity grades ranging from 0 = no chronic pain to 4 = high disability, severely limiting (see [20,21] for further details). Obtaining a CPG score of 3 or 4 was defined as non-response to treatment, because having a moderate to high level of pain-related disability following treatment indicates that the treatment goals of the IIPT were not met. Secondary outcomes were the individual pain characteristics, i.e., pain intensity and disability at T5. Univariate logistic regression analyses assessing whether the PSOCQ stage at T4 (treated as continuous) predicted non-response to treatment at T5 (short-term non-responders: CPG 3–4) were calculated to investigate whether the PSOCQ is suitable for predicting treatment outcome. In addition, a subsequent model assessing whether improvement in PSOCQ stage from T1 to T4 (yes/no) predicted treatment non-response was conducted. Equivalent analyses were conducted using linear regression models for each pain characteristic (pain intensity, pain-related disability). In these analyses, predictive validity would be indicated if lower odds of non-response and lower levels of pain-related outcomes followed more advanced stages of change at discharge [3,4]. Similarly, advancing change in stage across treatment was expected to be negatively associated with treatment failure, pain intensity and pain-related disability at the three-month follow-up.

## 3. Results

### 3.1. Study 1

#### 3.1.1. Factor Structure Prior to Intake (Sample A)

In Sample A, both the four-factor and the three-factor model of the PSOCQ-A yielded an inadequate model fit with a poor to acceptable fit to the data. The factor intercorrelation between the Action and Maintenance subscales was very high for the four-factor model (0.96). Cronbach’s α ranged from 0.59 to 0.89. Likewise, for the PSOCQ-P, an inadequate model fit was found for both the four-factor (poor to acceptable fit) and the three-factor (poor fit) model. Cronbach’s α ranged from 0.59 to 0.88. See Table 2 for an overview of all fit indices for all models tested. Deleting items with low factor loadings < 0.5 did not improve model fit.

#### 3.1.2. Factor Structure at Admission to IIPT (Sample B)

In Sample B, both the four-factor and three-factor models of the PSOCQ-A yielded an acceptable to good model fit. Only the CFI indicated poor fit. Because of a high factor correlation between the Action and Maintenance subscales (0.9), the three-factor solution with the combined Action/Maintenance subscale was preferred. Cronbach’s α was 0.60 for Precontemplation, 0.72 for Contemplation and 0.90 for Action/Maintenance. To reduce the questionnaire length, items with low factor loadings < 0.5 were deleted for the Precontemplation (items 11, 16, 24, 25) and Contemplation (items 7, 14, 19, 21, 23, 28) subscales. For the combined Action/Maintenance subscale, items with factor loadings < 0.7 (items 2, 4, 10, 13, 20, 26, 30) were deleted to have an approximately equal distribution of item numbers for the three subscales. Model fit of this shortened three-factor version was acceptable to good and resulted in a much-improved CFI, which was just below the cutoff for acceptable fit. Table 2 provides an overview of fit indices for all models tested. Cronbach’s α of these shortened subscales was 0.56 for Precontemplation, 0.65 for Contemplation and 0.89 for the combined Action/Maintenance subscale.

For the PSOCQ-P, the four-factor model yielded an acceptable to good model fit (CFI indicated poor fit) with all factor correlations < 0.6. The three-factor model resulted in a poor to acceptable model fit. Therefore, the four-factor solution was chosen as the preferred option. Cronbach’s α was 0.53 for Precontemplation, 0.68 for Contemplation, 0.73 for Action and 0.90 for Maintenance. Consistent with the PSOCQ-A, a shortened version of the PSOCQ-P was developed. Therefore, items with low factor loadings < 0.5 were deleted for the Precontemplation (items 11, 16, 22, 24, 25, 29), Contemplation (items 1, 7, 8, 21, 23, 28) and Action (item 26) subscales. There were no items with factor loadings < 0.5 for the Maintenance subscale. After the deletion of these items, the Precontemplation factor only consisted of one item. Thus, the Precontemplation factor was deleted. There was an approximately equal distribution of item numbers for the three remaining subscales Contemplation, Action, Maintenance. Model fit of this shortened three-factor version was acceptable to good. See Table 2 for an overview of fit indices for all models tested. Cronbach’s α of these shortened subscales was 0.75 for Contemplation, 0.76 for Action and 0.90 for Maintenance. The shortened versions of the PSOCQ-A and the PSOCQ-P can be found in Appendix A, Table A1.

### 3.2. Study 2

#### 3.2.1. Factor Structure and Internal Consistency

For the PSOCQ-A short-version, the three-factor model yielded an acceptable (RMSEA = 0.07, SRMR = 0.08) to good (χ^2^/df = 1.7) model fit. The CFI was just below the cutoff for acceptable fit (CFI = 0.93). The internal consistency was not sufficient for Precontemplation and Contemplation subscales (Cronbach’s α = 0.47 and 0.56, respectively), but was good for the combined Action/Maintenance subscale (Cronbach’s α = 0.90). Overall, an acceptable model fit was found for the PSOCQ-P three-factor model (χ^2^/df = 2.1, SRMR = 0.08). The RMSEA was just above the cutoff for acceptable fit (RMSEA = 0.09). The CFI indicated poor fit (CFI = 0.88). Internal consistency was acceptable to good for all subscales: Contemplation (Cronbach’s α = 0.71), Action (Cronbach’s α = 0.75) and Maintenance (Cronbach’s α = 0.90).

#### 3.2.2. Stage Assignment and Sensitivity to Change

**Figure 1:**
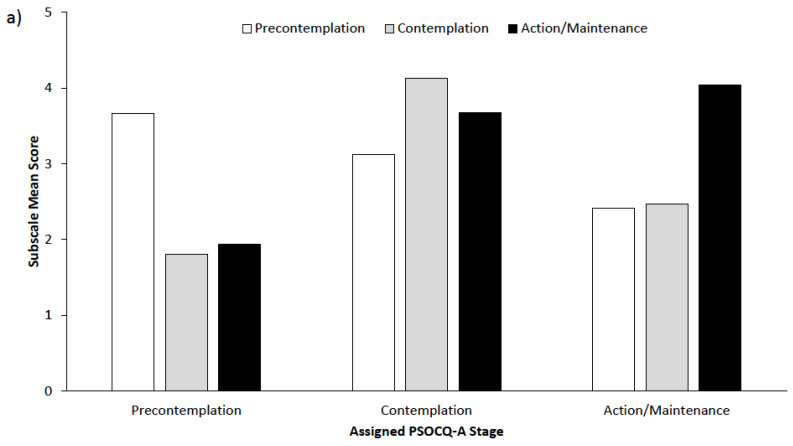

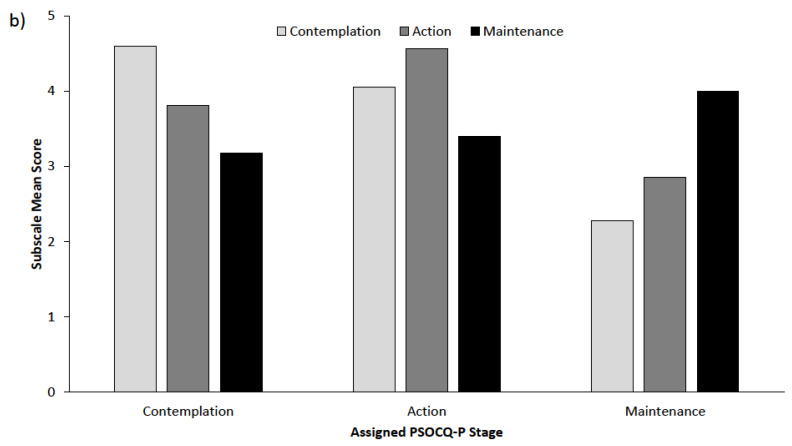
(**a**) Pain Stages of Change Questionnaire for Adolescents (PSOCQ-A) and (**b**) Pain Stages of Change Questionnaire for Parents (PSOCQ-P) subscale scores of the patients assigned to the different stages of change at admission. *Note*: Patients were assigned to one stage of change based on their highest subscale mean score at T1.

At T1, 2.7% of adolescents were categorized as being in the Precontemplation stage, 90% in the Contemplation stage (90.0%), and 7.3% in the Action/Maintenance stage. Regarding parents, 24.6% were categorized in the Contemplation stage, 70.4% were in the Action stage, and 4.9% were in the Maintenance stage at T1. Figure 1 displays the PSOCQ-A and PSOCQ-P profiles of the different stage groups. In adolescents categorized in the Precontemplation and Action/Maintenance stages (PSOCQ-A), their scores for the other stages tended to be much lower. However, for adolescents in the Contemplation stage, stage assignment was less clear, with relatively high mean scores for all stages. More variation was noted in the parent PSOCQ-P profiles.

For assessment of the PSOCQ-A, a Friedman test indicated a significant increase in stages of change over treatment (T1, T2, T3, T4; χ^2^ = 44.7, *p* < 0.001). From T1 to T4, 32.1% of the patients moved to a higher stage of change (primarily from Contemplation to Action/Maintenance), while 62.0% remained in the same stage and only 5.8% moved to a lower stage of change. Additionally, a significant increase was present from T4 (discharge) to T5 (3-month follow-up) (*p* = 0.021). 

**Figure 2:**
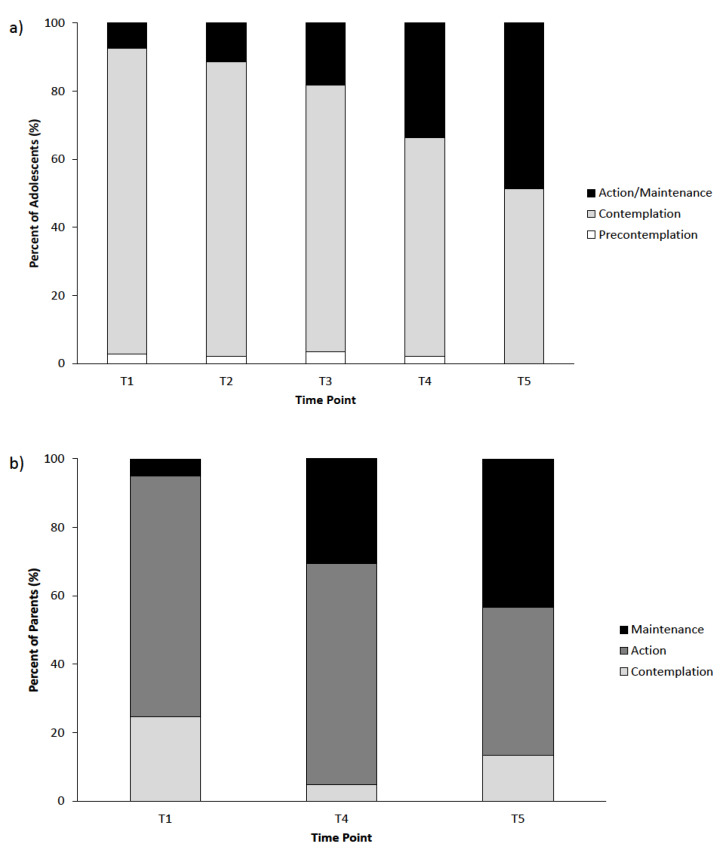
Frequency of stages of change over the course of treatment for (**a**) Pain Stages of Change Questionnaire for Adolescents (PSOCQ-A) and (**b**) Pain Stages of Change Questionnaire for Parents (PSOCQ-P). *Note*: T1 = Admission; T2 = 1 week after admission; T3 = 2 weeks after admission; T4 = Discharge (approx. 3 weeks after admission); T5 = 3 months after discharge. At each time point, patients were assigned to one stage of change according to their highest subscale mean score.

Assessment of the PSOCQ-P revealed that parents were in higher stages of change at discharge (T4) compared to admission (T1; Z = -5.88, *p* < 0.001). Approximately half of the parents (46.3%) moved to a higher stage of change from T1 to T4, while only 6.6% moved to a lower stage. There was no significant difference between stage assignment at T4 and T5 (*p* = 0.424). Figure 2 displays the stage assignment over time for both the PSOCQ-A and PSOCQ-P.

#### 3.2.3. Concurrent Criterion Validity: Association of Stage of Change with Pain Characteristics

Kruskal–Wallis tests conducted on adolescent admission (T1) data indicated significant differences between stage groups for maximum and average pain intensity (all *p* < 0.02). Post-hoc analyses revealed that patients in the Precontemplation stage reported significantly higher maximum and average pain intensity than patients in the Contemplation (all *p* < 0.01) and Action/Maintenance stages (all *p* < 0.02). There were no significant differences between the stage groups for pain-related disability, school absence or pain coping strategies. 

Kruskal–Wallis tests conducted on admission (T1) data assessing the impact of parental stage of change on adolescent pain-related outcomes indicated a significant difference between parental stage groups for adolescent positive self-instruction coping strategies (*p* = 0.036). Post-hoc analyses revealed that adolescents with parents in the Contemplation stage reported significantly lower positive self-instruction than patients with parents in the Maintenance stage (*p* = 0.042). There were no significant differences between the parental stage groups for adolescent pain intensity, pain-related disability, school absence or other types of pain coping strategies (passive pain coping, seeking social support). 

#### 3.2.4. Predictive Criterion Validity: Association of Stage of Change with Non-Response to Treatment

Our primary treatment-related outcome was whether adolescents responded to treatment. Overall, 80.3% of all patients were categorized as responders and 19.7% were categorized as non-responders to treatment at T5 (three months after treatment). Logistic regression analyses found no significant associations per PSOCQ stage of change as measured by PSOCQ-A or PSOCQ-P at T4 (discharge) and the treatment non-response outcome, nor presence of stage improvement in adolescents or parents over the course of treatment with non-response (see Table 3).

The prediction of secondary treatment outcomes, i.e., pain intensity or pain-related disability three months post-treatment, by stage of change at treatment discharge, were analyzed. Linear regression models revealed no significant associations between stage of change as measured by PSOCQ-A at T4 (discharge) and maximum pain intensity, average pain intensity or pain-related disability at T5. When conducting linear regression analyses with the predictor variable of whether improvement in adolescent stage of change occurred during treatment, average intensity had a significant relationship, in which an improvement in stage of change during treatment was associated with a reduction of 0.87 points on the NRS, (*p* = 0.037). No association with maximum intensity or pain-related disability was found. The parent stage of change at T4 and improvement in parental stage of change occurring during treatment were not associated with pain characteristics in adolescents three-months post-discharge.

## 4. Discussion

The aim of the present study was to create and validate German versions of the PSOCQ-A and the PSOCQ-P. In Study 1, a short version was created that was cross-validated in Study 2. An acceptable to good model fit was found for both PSOCQ-A and PSOCQ-P. However, internal consistency scores for Precontemplation and Contemplation scales were low for the PSOCQ-A. The results also indicated that it may not be useful to administer the PSOCQ-A before initial presentation to specialized pain care. Readiness to change was associated with pain intensity in the present study, but not with functional impairment, pain coping or with treatment failure three months after discharge. According to both patient- and parent-report, willingness to change appeared to progress over the course of treatment.

Regarding the PSOCQ-A, we confirmed a factor structure consistent with previous studies (Precontemplation, Contemplation, Action/Maintenance) [2,18]. For the PSOCQ-P, previous studies have found support for a four-factor solution (Precontemplation, Contemplation, Action, Maintenance) [2,18]. We similarly found support for a four-factor solution when including all original questionnaire items. However, these findings were only evident in patients and parents admitted to inpatient IIPT. In the group of adolescents and parents completing the questionnaire prior to adolescent intake at a specialized pain treatment center, model fit was poor.

The problematic factor structure of the PSOCQ-A and PSOCQ-P in individuals completing the questionnaires prior to intake may be due to these patients and parents being unfamiliar with a self-management approach to pain, as noted by intake staff. This may have caused difficulty answering items using the scale of ‘strongly disagree’ to ‘strongly agree’ for items indicative of later stages of change (e.g., “When my pain flares up, I find myself using coping strategies that have worked in the past, such as a relaxation exercise or distractions.”; “I use what I have learned to help keep my pain under control”). Contrastingly, in the original English validation study, patients and their parents completed the PSOCQ-A and the PSOCQ-P prior to initial evaluation in a similar setting to ours, with no difficulties noted by researchers [2]. Usually patients are referred to specialized centers from other medical subspecialties because previous treatments have failed [2,22]. It is not clear how much information has been provided to these patients about pain self-management before presentation to specialized care. Differences in health care systems, or available chronic pain education outside of treatment settings, may be responsible for the discrepant findings.

A German pilot study revealed low acceptance of the PSOCQ questionnaires [10]. In response, we created short versions of the PSOCQ-A and PSOCQ-P in Study 1 in an attempt to make accurate completion more likely. However, these revised versions lost comparability with the original version. Furthermore, given that the PSOCQ-A resulted in Precontemplation, Contemplation and Action/Maintenance factors, and the PSOCQ-P resulted in Contemplation, Action and Maintenance factors, the parent and child version are no longer directly comparable. Therefore, addressing one issue resulted in other problems in the validity of this tool.

Other psychometric issues for the PSOCQ-A were also apparent in our study, with low (≤0.60) internal consistency found in both the original and shortened versions of the Precontemplation subscale. The Precontemplation subscale of the shortened PSOCQ-A version, in particular, had only three items and very low internal consistency, and therefore should be used with caution. Previous studies have consistently found the lowest internal consistency scores for the Precontemplation subscale [2,3,18,23]. Additionally, the value of the Precontemplation subscale in the pediatric chronic pain population is questionable, as only 2.7% of adolescent pain patients in this stage were at the start of in-patient treatment in the present study. This distribution is in sharp contrast to previous studies with adolescents, where one third to one half of the participants were categorized as being in the Precontemplation stage prior to intake [2,3,18,23]. Some important methodological differences should be noted here, with our sample being collected during their admission to in-patient pain treatment, whereas all four pediatric studies were collected prior to intake (with three out of four through mail-out questionnaires). However, in adult studies assessing the stage of change at a similar stage of treatment to our sample, Jensen et al. [24] and Strong et al. [25] found that only a small number of adult patients were categorized in Precontemplation, and that most patients were in the Contemplation stage. They concluded that only patients who are motivated and ready to change complete the intake procedures and are admitted for specialized treatment [24,25]. This indicates that the Precontemplation subscale in the PSOCQ-A may have some value prior to treatment, however, once admission to specialist treatment has been confirmed, this subscale no longer appears to have much clinical utility. 

Despite difficulties with factor structure and low internal consistencies, change in stages across treatment, as measured by the PSOCQ-A and the PSOCQ-P, was in the expected directions and reflected treatment progress. Logan et al. [3] and Sherry et al. [26] also demonstrated significant changes in readiness to change during IIPT using the PSOCQ-A and PSOCQ-P. In combination, evidence suggests that interdisciplinary pediatric pain treatment increases readiness to change [3], with the PSOCQ able to monitor this component of treatment progress.

The assessment of criterion validity in the PSOCQ-A, in line with findings by Guite et al. [2], identified significant associations between pain intensity and readiness to change stage, in which higher pain intensity was found in adolescents in the Precontemplation stage. Other studies have additionally found significant associations between readiness to change stage and other pain characteristics, such as pain-related disability or passive pain coping [2,18], that were not replicated in the present study. Important methodological differences should be noted here. While other studies assessed correlations between subscale scores and outcome scores, here we assessed for statistical differences between groups defined by their stage category. As the aim of the PSOCQ is to identify the stage of change at a given time, the observation of between-group differences is an important component of validity. Our study suggests limited concurrent criterion validity of the PSOCQ-A.

Similar assessments of parental stages of change through the PSOCQ-P identified associations with child coping, but not pain-related outcomes. This is consistent with other research identifying that adolescent psychological components were inversely associated with parental stage of change, including fear of pain [3], pain catastrophizing [18,23] and anxiety [18], although these were not assessed in this study. Parental stage of change was associated with adolescent pain in one study. In this case, the Precontemplation stage alone was associated with adolescent higher pain-related disability [2]. Generally, our results indicated some level of consistency regarding the concurrent criterion validity of the PSOCQ-A and PSOCP-P, however this was not a strong finding when assessing individual stages of change with outcomes.

Limited predictive criterion validity was found in our study. Consistent with cross-sectional associations with pain intensity, we found that in adolescents, transferring to a higher stage of change during treatment was associated with a reduction in their pain intensity post-treatment, however, did not find that readiness to change was a significant predictor of disability or treatment failure, nor did we find that stage of change at the end of treatment was associated with post-treatment outcomes. Furthermore, pretreatment scores could not be used for prediction in this study due to low variance, because most adolescent patients were in the same (Contemplation) stage. A study with similar methodology found somewhat consistent results. Logan et al. reported that increases in readiness to change over treatment were associated with decreases in functional disability and maladaptive psychological outcomes [3]. The authors reported very few meaningful associations with treatment outcomes were found for individual stages of change assessed at pre-treatment. In combination, these findings suggest the utility of the tool may be in tracking mindset shift as identified by a change in stage of change, rather than identifying a particular stage. 

### 4.1. Clinical Implications

Given that the PSOCQ-A and PSOCQ-P were developed and validated in the United States, it was important for us to ensure psychometric properties were maintained in the German translation, particularly as findings from the German pilot study indicated that the population had difficulty interpreting items. Currently, given our findings, we do not recommend using the PSOCQ-A and PSOCQ-P as a measure of the stage of change. This is for a few reasons: validity was not achieved in a pre-clinical sample; associations between stages of change and pain-related outcomes tended to be seen with regard to changes across stages, rather than regarding specific stages themselves; and treatment failure was not predicted. 

### 4.2. Strengths and Limitations

The strengths of this study are the large sample in Study 1, the application of the survey in different settings (outpatient vs. inpatient), cross-sectional and longitudinal validation, and the focus on improving the usability of the PSOCQ in clinical practice. However, a focus on clinical utility resulting in the shortening of the questionnaires limits the comparability of the German versions with other studies. Furthermore, the German versions have only been tested in a single pain-treatment center. 

## 5. Conclusions

Issues were noted when using the PSOCQ in patients not yet familiar with chronic pain and its treatment approach, with validity improving when used with a pain sample at admission to specialized pain treatment, who had already been provided with some education about these topics. German short versions of the PSOCQ-A and the PSOCQ-P were developed in the present study. The results indicate that the German PSOCQ-A and PSOCQ-P might be somewhat useful for monitoring treatment progress but can generally not be recommended to assess pain stages of change or to predict treatment outcome.

## Figures and Tables

**Table 1:** Characteristics of the three adolescent study samples.

	Study 1		Study 2
	Sample A (*N* = 501) ^a^	Sample B (*N* = 240) ^a^	(*N* = 150) ^a^
*Demographics*			
Sex (female), *N* (%)	353 (70.5)	164 (68.3)	105 (70.0)
Age (years), Mean (SD)	14.4 (1.8)	14.5 (1.8)	14.4 (1.9)
*Pain characteristics*			
Maximum pain intensity ^b^, Mean (SD)	8.2 (1.5)	7.5 (1.9)	8.3 (1.4)
Average pain intensity ^b^, Mean (SD)	6.5 (1.8)	5.9 (2.1)	6.0 (2.0)
Pain duration (months), Mean (SD)	34.5 (37.9)	40.6 (39.5)	28.6 (28.2)
Pain-related school absence ^c^, Mean (SD)	2.9 (3.9)	4.5 (6.7)	3.9 (5.7)
Pain-related disability ^d^, Mean (SD)	36.1 (9.5)	32.3 (9.3)	34.8 (8.6)
*Primary pain location*			
Head, *N* (%)	254 (51.2)	110 (47.0)	64 (43.0)
Abdomen, *N* (%)	74 (14.9)	30 (12.8)	22 (14.8)
Musculoskeletal system, *N* (%)	107 (21.6)	58 (24.8)	38 (25.5)
Other, *N* (%)	12 (2.4)	7 (3.0)	2 (1.3)
>1 primary pain location, *N* (%)	49 (9.9)	29 (12.4)	23 (15.4)

*Note*: ^a^*n* varies due to missing values. ^b^ Numerical Rating Scale (Range: 0–10). ^c^ days in the last 4 weeks (Range: 0–20). ^d^ Pediatric Pain Disability Index (Range: 12–60).

**Table 2:** Model fit indices of all models tested.

	χ^2^	df	χ^2^/df ^a^	CFI ^b^	RMSEA ^c^	SRMR ^d^
*PSOCQ-A*						
Sample A						
4-factor model (P, C, A, M)	1349.9	399	3.4	0.79	0.07	0.08
3-factor model (P, C, A/M)	1394.4	402	3.5	0.79	0.07	0.08
Sample B						
4-factor model (P, C, A, M)	759.0	399	1.9	0.83	0.06	0.08
3-factor model (P, C, A/M)	797.7	402	2.0	0.82	0.06	0.08
3-factor model short version (P, C, A/M)	121.1	62	2.0	0.94	0.06	0.06
*PSOCQ-P*						
Sample A						
4-factor model (P, C, A, M)	1232.8	399	3.1	0.76	0.07	0.08
3-factor model (P, C, A/M)	1775.9	402	4.4	0.61	0.09	0.12
Sample B						
4-factor model (P, C, A, M)	790.1	399	2.0	0.80	0.07	0.08
3-factor model (P, C, A/M)	1117.2	402	2.8	0.64	0.09	0.12
3-factor model short version (C, A, M)	181.1	101	1.8	0.95	0.06	0.07

*Note*: PSOCQ-A = Pain Stages of Change Questionnaire for Adolescents. PSOCQ-P = Pain Stages of Change Questionnaire for Parents. Subscales: P = Precontemplation, C = Contemplation, A = Action, M = Maintenance. ^a^ ≤3 = acceptable, ≤2 = good. ^b^ Comparative Fit Index; ≥0.95 = acceptable, ≥0.97 = good. ^c^ Root Mean Square Error of Approximation; ≤0.08 = acceptable, ≤0.05 = good. ^d^ Standardized Root Mean Square Residual; ≤0.10 = acceptable, ≤0.05 = good.

**Table 3:** Results of the univariate logistic regression analyses predicting non-response to treatment three months after discharge.

Predictor	Odds Ratio	95% CI	*p*
*Adolescents (n = 108)*			
PSOCQ-A stage improved during treatment (T1–T4)	0.70	0.25–1.98	0.501
Per increase in stage of PSOCQ-A at T4 ^a^	0.66	0.26–1.66	0.379
*Parents (n = 103)*			
PSOCQ-P stage improved during treatment (T1–T4)	0.62	0.23–1.65	0.336
Per increase in stage of PSOCQ-P at T4 ^a^	1.10	0.47–2.60	0.828

*Note*: PSOCQ-A = Pain Stages of Change Questionnaire for Adolescents. PSOCQ-P = Pain Stages of Change Questionnaire for Parents. CI = confidence interval. T1 = admission; T4 = discharge. ^a^ Given the small participant number in the Precontemplation Stage of the PSOCQ-A and Contemplation Stage of the PSOCQ-P at T4, this variable was treated as continuous rather than categorical.

## Appendix A

**Table A1:** Short versions of the PSOCQ-A and the PSOCQ-P.

Original Item Number	Item Description	Subscale
*PSOCQ-A*		
1	Ich denke schon länger darüber nach, dass mein Umgang mit Schmerzen besser werden könnte. (I have been thinking that the way I cope with my pain could get better.)	Contemplation
3	Ich habe einige gute Strategien gelernt, damit mein Schmerzproblem nicht mein Leben beeinträchtigt. (I have learned some good ways to keep my pain problem from getting in the way of my life.)	Action/ Maintenance
5	Ich wende einige Strategien an, die mir helfen, im Alltag besser mit meinen Schmerzen umzugehen. (I am using some strategies that help me better deal with my pain on a day-to-day basis.)	Action/ Maintenance
6	Ich habe begonnen, Strategien zu entwickeln, um meine Schmerzen zu kontrollieren. (I have started to come up with strategies to help myself control my pain.)	Action/ Maintenance
8	Selbst wenn meine Schmerzen nicht vollständig weggehen, bin ich bereit, meinen Umgang mit den Schmerzen zu verändern. (Even if my pain doesn’t go away, I am ready to start changing how I deal with it.)	Contemplation
9	Ich weiß jetzt, dass es an der Zeit ist, einen besseren Umgang mit meinen Schmerzen zu entwickeln. (I realize now that it’s time for me to come up with a better plan to cope with my pain problem.)	Contemplation
12	Ich glaube, meine Schmerzen sind ein medizinisches Problem und ich sollte mich deshalb ausschließlich von Ärzten behandeln lassen. (My pain is a medical problem and I should be dealing with medical doctors about it.)	Precontemplation
15	Ich habe kürzlich erkannt, dass vor allem ich selber etwas für einen besseren Umgang mit meinen Schmerzen tun kann. (I have recently figured out that it’s up to me to deal better with my pain.)	Contemplation
17	Ich habe Schmerzbewältigungsstrategien entwickelt, die ich in meinem Alltag nutze. (I have built strategies for dealing with my pain into my everyday life.)	Action/ Maintenance
18	Ich habe große Fortschritte im Umgang mit meinen Schmerzen gemacht. (I have made a lot of progress in coping with my pain.)	Action/ Maintenance
22	Obwohl Ärzte mir etwas anderes sagen, denke ich noch immer, dass meine Schmerzen mit Hilfe einer Operation oder durch Medikamente verschwinden können. (I still think despite what doctors tell me, there must be some surgery or medicine that would get rid of my pain.)	Precontemplation
27	Ich probiere einige Bewältigungsstrategien aus, um meine Schmerzen besser in den Griff zu bekommen. (I am testing out some coping skills to manage my pain better.)	Action/ Maintenance
29	All das Gerede, wie man besser mit Schmerzen umgehen kann, ist für mich Zeitverschwendung. (All of this talk about how to cope better is a waste of my time.)	Precontemplation
*PSOCQ-P*		
2	Ich ermutige mein Kind, neue Strategien im Umgang mit seinen/ihren Schmerzen zu entwickeln. (I am encouraging my child to develop new ways to cope with his/her pain.)	Action
3	Mein Kind hat einige gute Strategien gelernt, damit das Schmerzproblem nicht sein/ihr Leben beeinträchtigt. (My child has learned some good ways to keep his/her pain problem from interfering with life.)	Maintenance
4	Wenn Schmerzen auftreten, wendet mein Kind sofort Bewältigungsstrategien an, die ihm/ihr in der Vergangenheit geholfen haben, z.B. Entspannungsübungen oder Ablenkung. (When my child’s pain flares up, s/he is automatically using coping strategies that have worked in the past, such as a relaxation exercise or a mental distraction technique.)	Maintenance
5	Mein Kind wendet einige Strategien an, die ihm/ihr helfen, im Alltag besser mit den Schmerzen umzugehen. (My child is using some strategies that help him/her better deal with his/her pain problem on a day-to-day basis.)	Maintenance
6	Ich ermutige mein Kind, Strategien zu entwickeln, um seine/ihre Schmerzen zu kontrollieren. (I am encouraging my child to come up with strategies to help him/her control pain.)	Action
9	Ich weiß jetzt, dass es an der Zeit ist, einen besseren Umgang mit den Schmerzen meines Kindes zu entwickeln. (I realize now that it’s time to come up with a better plan to cope with my child’s pain problem.)	Contemplation
10	Mein Kind wendet das an, was er/sie gelernt hat, um die Schmerzen in den Griff zu bekommen. (My child uses what s/he has learned to help keep his/her pain under control.)	Maintenance
13	Mein Kind setzt aktuell einige Empfehlungen um, damit er/sie mit den Schmerzen leben kann. (My child is currently using some suggestions people have made about how to live with his/her pain problem.)	Maintenance
14	Seit Kurzem frage ich mich, ob mein Kind Hilfe benötigt, Strategien zu entwickeln, die ihm/ihr im Umgang mit den Schmerzen helfen. (I am beginning to wonder if my child needs to get some help to develop skills for dealing with his/her pain.)	Contemplation
15	Ich habe kürzlich erkannt, dass vor allem mein Kind selber etwas für einen besseren Umgang mit den Schmerzen tun kann. (I have recently figured out that it’s up to my child to deal better with his/her pain.)	Contemplation
17	Mein Kind hat Schmerzbewältigungsstrategien entwickelt, die er/sie im Alltag nutzen kann. (My child has incorporated strategies for dealing with pain into his/her everyday life.)	Maintenance
18	Mein Kind hat große Fortschritte im Umgang mit den Schmerzen gemacht. (My child has made a lot of progress in coping with his/her pain.)	Maintenance
19	Ich habe kürzlich erkannt, dass mein Kind den Umgang mit den Schmerzen verändern muss. (I have recently come to the conclusion that it’s time for my child to change how s/he copes with his/her pain.)	Contemplation
20	Ich ermutige mein Kind Unterstützung in Anspruch zu nehmen, um Strategien für einen besseren Umgang mit den Schmerzen zu erlernen. (I am encouraging my child to get help with learning some strategies for coping better with his/her pain.)	Action
27	Ich ermutige mein Kind, einige Bewältigungsstrategien auszuprobieren, um die Schmerzen besser in den Griff zu bekommen. (I am encouraging my child to test out some coping skills to manage his/her pain better.)	Action
30	Ich ermutige mein Kind andere Möglichkeiten als Medikamente und Operationen kennenzulernen, um die Schmerzen in den Griff zu bekommen. (I am encouraging my child to learn ways to control his/her pain other than with medications or surgery.)	Action
